# The lung–gut axis during viral respiratory infections: the impact of gut dysbiosis on secondary disease outcomes

**DOI:** 10.1038/s41385-020-00361-8

**Published:** 2021-01-26

**Authors:** Valentin Sencio, Marina Gomes Machado, François Trottein

**Affiliations:** 1 US 41-UMS 2014-PLBS, U1019-UMR 9017-CIIL—Centre d’Infection et d’Immunité de Lille, Univ. Lille, F-59000 Lille, France; 2 Centre National de la Recherche Scientifique, UMR 9017, F-59000 Lille, France; 3 Institut National de la Santé et de la Recherche Médicale, U1019, F-59000 Lille, France; 4 Centre Hospitalier Universitaire de Lille, F-59000 Lille, France; 5 Institut Pasteur de Lille, F-59000 Lille, France

## Abstract

Bacteria that colonize the human gastrointestinal tract are essential for good health. The gut microbiota has a critical role in pulmonary immunity and host’s defense against viral respiratory infections. The gut microbiota’s composition and function can be profoundly affected in many disease settings, including acute infections, and these changes can aggravate the severity of the disease. Here, we discuss mechanisms by which the gut microbiota arms the lung to control viral respiratory infections. We summarize the impact of viral respiratory infections on the gut microbiota and discuss the potential mechanisms leading to alterations of gut microbiota’s composition and functions. We also discuss the effects of gut microbial imbalance on disease outcomes, including gastrointestinal disorders and secondary bacterial infections. Lastly, we discuss the potential role of the lung–gut axis in coronavirus disease 2019.

## Introduction

The gastrointestinal tract hosts a complex, highly diverse microbial ecosystem (mostly obligate anaerobic bacteria) that is commonly referred to as the gut microbiota. Collectively, intestinal bacteria constitute a genome of more than 3 million genes—150 times larger than the human genome.^1^ The gut microbiota is dominated by the Firmicutes (e.g., *Lactobacillus*, *Bacillus*, and *Clostridium*), the Bacteroidetes (e.g., *Bacteroides*), and to a lesser extent, the Proteobacteria (e.g., *Escherichia*) and the Actinobacteria (e.g., *Bifidobacterium*). Experimental and clinical evidences demonstrate that the gut microbiota has a crucial role in the maintenance of human health.^2^ Tightly regulated microbiota-host interplay influences the development, instruction, and priming of the immune system.^3–6^ Recent research studies have shown that this interplay has a major role in the control of infection.^7^ The mechanism by which the gut microbiota regulates immune responses depends on microbial-associated molecular patterns, microbial metabolites, and the microbes’ interactions with progenitor cells and mature immune cells.^8–10^

Many factors can alter the diversity and composition of the gut microbiota, leading to dysbiosis. This includes dietary patterns and stress inducers, such as broad-spectrum antibiotics and cancer drugs. Disease situations—such as infections and chronic inflammatory or metabolic disorders—can also lead to dysbiosis.^11^ Changes in intestinal bacterial communities can influence disease outcomes even in distant organs (including the lungs), as demonstrated by transfer experiments with dysbiotic microbiota.^8–11^ Here, we summarize mechanisms through which the gut microbiota remotely promotes pulmonary defense against viral infection. We review the effects of acute viral respiratory infections on the gut microbiota, discuss the potential mechanisms of gut dysbiosis and debate the impact of altered gut microbiota on secondary disease outcomes. The potential importance of the gut–lung axis in the outcome of coronavirus disease-19 (COVID-19) is discussed in the light of this knowledge.

## The gut microbiota in the lung’s defenses against viral respiratory infections

The role of the gut microbiota in resistance to colonization to enteric pathogens is well established.^7^ Accordingly, alterations of the microbiota notably induced by antibiotics, which eradicate more or less completely intestinal bacteria or specific species, increase susceptibility to enteric infections like *Salmonella* and enteroaggregative *Escherichia coli.*^3,4,6,11^ This beneficial effect of the gut microbiota in immunity is not limited to the gut compartment but also extends to systemic compartments and distant organs such as the lungs.^12,13^ Mice lacking microbiota (i.e., germ free) or those orally treated with antibiotics (broad spectrum or targeted antibiotics such as neomycin, metronidazole or vancomycin) have impaired responses to systemic and respiratory infections.^14–25^ In the setting of viral respiratory infections caused by, for example, influenza A virus (IAV) and respiratory syncytial virus (RSV), the profound disturbance of the gut microbiota’s ecology by antibiotic therapy weakens the host’s innate and adaptive defenses.^14–18,26^ Mechanisms through which the gut microbiota arms the lung to control viral respiratory infection are numerous. These points will not be extensively reviewed in the present review but some examples will be provided (for reviews,^27–30^). Briefly, several studies have highlighted the impact of the gut microbiota on the lung’s production of type I interferons (IFNs),^15,16,18,31^ which are well known to control viral infections including severe acute respiratory syndrome coronavirus 2 (SARS-CoV-2), the etiological agent of COVID-19.^32–34^ In this setting, microbial metabolites such as desaminotyrosine (derived from flavonoid and amino acid metabolism) and short chain fatty acids (SCFAs, the end products of dietary fiber fermentation by commensal bacteria) have been shown to be critical.^15,18^ For example, it was shown that desaminotyrosine, produced by an obligate clostridial anaerobe (*Clostridium orbiscindens*, metronidazole, and vancomycin sensitive) from the digestion of plant flavonoids, could diffuse into blood, reach the lungs and prime the (innate) immune system to protect from influenza infection.^18^ Desaminotyrosine promoted the synthesis of IFN-stimulated genes in lungs and pulmonary phagocytes were critical in this mechanism. In this setting, desaminotyrosine augmented type I IFN signaling by IFN amplification via IFN-α/β receptor and signal transducer and activator of transcription 1.^18^ For SCFAs, a myriad of commensals, including members of the Lachnospiraceae family, are able to ferment dietary fibers. For example, it was shown that acetate could diffuse into blood and activate the G protein coupled receptor (GPR) 43 (also termed free fatty acid receptor 2) expressed by stromal cells. This activating pathway improved type I IFN responses and increased IFN-stimulated gene expression.^15^ The mechanism through which GPR43 promoted type I IFN-mediated protection from influenza infection needs to be elucidated. Along with microbial metabolites, microbial membrane components derived from intact gut microbiota, including Toll-like receptor (TLR) ligands, also play a critical part in host defense against viral respiratory infections. Indeed, local and distal inoculation of Poly (I:C) (TLR3 agonist) or peptidoglycan (TLR2 agonist) rescued the immune impairment in the antibiotic-treated mice.^17^ It is noteworthy that in addition to these distal signals emanating from the gut microbiota, local microbes from the upper airway also regulate lower airway immunity and play a part in pulmonary defense against viral infections. The mechanisms have been described and/or reviewed elsewhere^24,29^ and will not be discussed in this review. The gut microbiota not only affects the innate immune response but also boosts CD8^+^ T-cell effector function—a process that is also involved in viral (influenza) clearance.^17,25,31^ As described above, altering the gut microbiota with antibiotics increased the severity of viral respiratory infections such as influenza. On the contrary, stimulating the microbiome with a high-fiber diet had the opposite effect.^15,31^ In the case of influenza, a high-fiber (fermentable inulin) diet conveyed protection through two distinct pathways. Fermentation of dietary fiber resulted in altered bone marrow hematopoiesis, leading to accumulation of alternatively activated macrophages in the lung of IAV-infected mice.^31^ These macrophages produced less chemokine (C-X-C motif) ligand 1, thus lowering early neutrophil infiltration into the airways. This pathway profoundly reduced exaggerated pulmonary inflammation and damage. The diet also boosted CD8^+^ T-cell metabolism and enhanced the effector functions of CD8^+^ T cells, effectively enhancing viral clearance. The GPR41 (also termed free fatty acid receptor 3) was required for the protective effect, and oral administration of SCFAs (butyrate) was sufficient to confer protection.^31^ Similar protective effects have been observed during RSV infection.^15^ A high-fiber (fermentable pectin) diet protected against RSV infection by modulating type I IFN response in lung epithelial cells and by increasing expression of interferon-stimulated genes in the lung. The GPR43 was required for the protective effect, and oral administration of acetate was sufficient to confer protection.^15^

The nature of gut commensal bacteria that exert antiviral effect in the lungs is still elusive. As discussed above, members of clostridial anaerobes (desaminotyrosine) and SCFA producers are important in the priming of the pulmonary innate immune system.^15,18^ More recently, a study based on the comparison of susceptible and resistant animals demonstrated that colonization by *Bifidobacterium* genus (*Bifidobacterium pseudolongum* and *Bifidibacterium animalis*) is strongly associated with the survival of influenza-infected mice.^35^ It is likely that in a near future, other commensal members will be identified and will serve as novel biomarkers to predict the severity and mortality of patients experiencing severe viral respiratory infections.

Hence, at steady state, resident bacteria from the gut microbiota can naturally and remotely buttress the lungs to combat viral respiratory infections. It has yet to be determined whether the gut microbiota is also involved in the early control (innate immunity) and late control (adaptive immunity) of infections by coronaviruses like SARS-CoV-2. The effect of gut microbiota depletion (by means of antibiotics) on the outcome of SARS-CoV-2 infection awaits further studies.

**Fig. 1:**
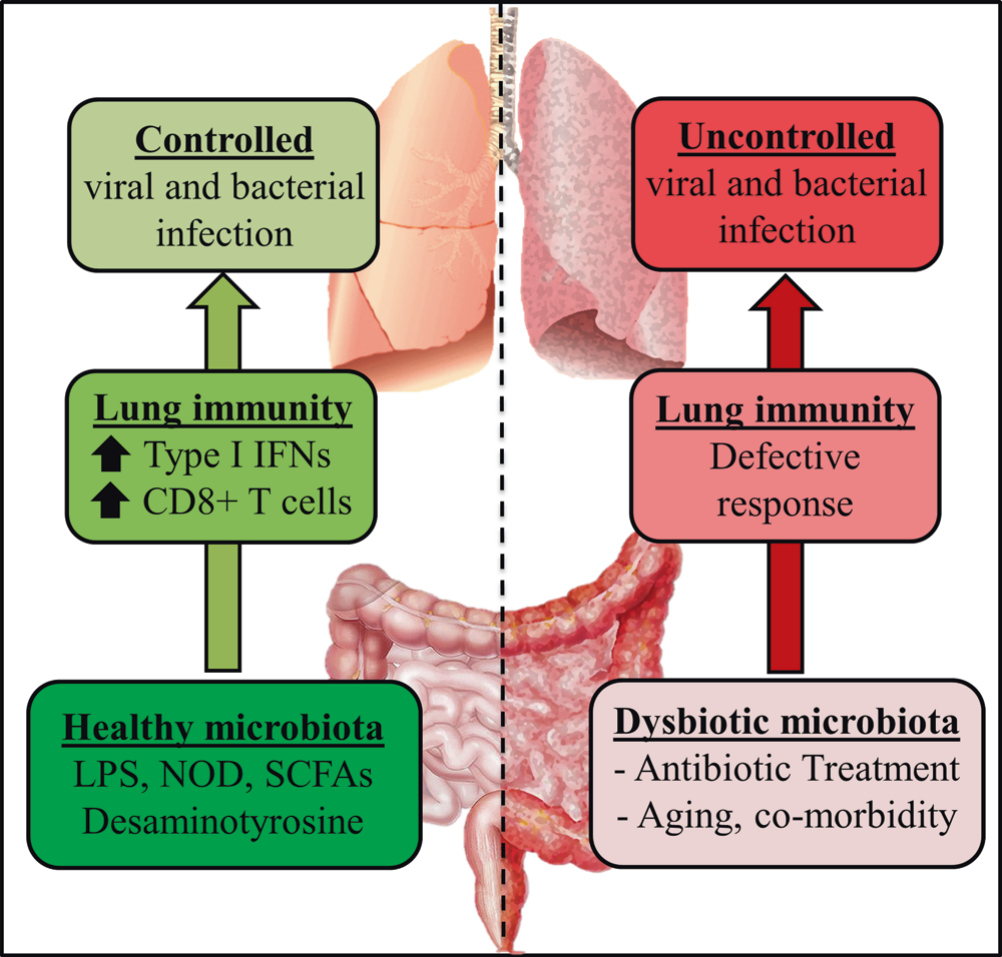
The role of the gut microbiota in viral respiratory infections. Factors released by a healthy gut microbiota arm the lungs against viral respiratory infections. In contrast, antibiotic treatment disturbs the ecological equilibrium in the gut and alters the lung’s defenses. Aging and co-morbidities are also associated with altered gut microbiota function and enhanced susceptibility to respiratory infections.

Regarding the role of the gut microbiota in lung’s defenses against respiratory viruses, it is plausible that any alteration in the microbiota composition and function can alter the beneficial cross-talk between the gut and the lungs (Fig. 1). Among factors and conditions involved in the alteration of the gut microbiota are the use of antibiotics, acute and chronic diseases, and aging. Co-morbidities such as obesity, diabetes and chronic respiratory diseases as well as aging are associated with perturbation (e.g., lower diversity) in gut microbiota composition and functionality.^6,36,37^ In these situations, it is possible that the dysfunctional gut microbiota distally influences pulmonary defenses against respiratory viruses. Fecal microbiota transplantation experiments (e.g., transfer of an aged-like gut microbiota into young mice) will be necessary to demonstrate this.

## Impact of viral respiratory tract infections on the gut microbiota

The “gut–lung axis” is bidirectional and pulmonary diseases (including infections) can influence the composition of the gut microbiota. Over the last few years, many researchers have used animal models of lung infections to analyse the effects of respiratory viruses (mostly IAV and RSV) on the gut microbiota. The impact of viral respiratory infections on the lung’s own microbiota is out of the scope of the present review.

### Influenza infection and gut microbiota

Influenza infections are responsible for significant morbidity and mortality, with 3–5 million severe cases and 290,000–650,000 deaths each year worldwide.^38^ Influenza can also cause devastating pandemics; the 1918 and 2009 pandemics killed 50 million people and 250,000–500,000 additional people, respectively.^39–41^ Influenza infections can lead to mild to severe pneumonia, acute respiratory distress syndrome (ARDS), clinically defined as acute respiratory failure, and multi-organ dysfunction.^38–40,42^ Despite the apparent absence of virus in the gut, some patients nevertheless present gastroenteritis-like symptoms such as abdominal pain, nausea, vomiting, and diarrhea.^43^ This suggests that the lung–gut axis is potentially important during influenza infection. Several research groups have studied the nature of microbial changes in the context of IAV infection in the mouse, mostly by 16S rRNA sequencing.^35,44–49^ Several IAV subtypes (H1N1, H3N2, H5N1, and H7N9) and sites, including the small intestine, cecum, and colon, have been studied. The data are convergent, i.e., alteration of the gut microbiota’s composition peaks at days 7–9 post infection. No virus was detected in the gut, and no change in the gut microbiota’s composition was observed after inoculation of an attenuated virus.^46^ The latter finding suggested that the change in the gut microbiota was specifically driven by a live viral infection and that the signal was not directly due to local replication. Alteration of the gut microbiota’s composition was transient and, although differences were still present, the composition globally returned to its initial state by 14 days post infection.^47,49^ The bacterial load and alpha diversity, as assessed by the number of operational taxonomic units, were not strongly impacted by IAV infection.^47,49^ At the phylum level, few changes were observed. Infections by the H1N1 subtype of IAV led to a decrease in the Bacteroidetes/Firmicutes ratio; this was mainly due to an increase in the *S24-7* (also known as *Muribaculaceae*) and *Porphyromonadaceae* families^46^ (Table 1). In contrast, H3N2 and H5N1 infections triggered a reduction in the *S24-7* family.^47,49^ An increase in Verrucomicrobia (mainly composed by the *Akkermansia* genus) was also observed during infection by H3N2 and H1N1 subtypes.^44,47^ These bacteria are known to degrade the intestinal mucus layer—as does *Ruminococcus*, numbers of which also increased during an IAV infection. A decrease in Actinobacteria (mainly due to reduction in *Bifidobacteriaceae* genus) has also been observed after IAV infection.^47^ Broader changes have been observed at lower taxonomic levels. Briefly, most studies found an increase in *Gammaproteobacteria* (potentially pathogenic *Escherichia coli*) and, less frequently, a decrease in the *Lactobacillus* genius (the *Bacilli* class)^45,47–49^ (Table 1). Microbiota analysis has also highlighted changes inside the Firmicutes phylum. In summary, the *Clostridiales* (unaffiliated), *Ruminococcaceae*, and *Mogibacteriacecea* families and the *Coprococcus*, *Roseburia*, *Defluvittalea*, *Dorea*, *Ruminococcus*, and *Gemmiger* genera were relatively more abundant during infection.^47^ Furthermore, IAV infection in mice was associated with a drop in the proportion of segmented filamentous bacteria (*Clostridiaceae* family).^45,48^ This bacterium interacts closely with the intestinal epithelium and is important in host resistance against enteric pathogens like *Citrobacter rodentium.*^50^ Taken as a whole, the data from murine models of influenza indicated that infection promotes the emergence of potentially detrimental bacterial species, such as members of *Gammaproteobacteria* and mucus-degrading bacteria. Conversely, infection appears to blunt the growth of health-promoting bacteria such as Lactobacilli, Bifidobacteria, and segmented filamentous bacteria.^48^ Interestingly, a recent study showed that the nature of the gut microbiota alteration due to IAV (H7N9) infection is different in mice that survived infection relative to mice that succumbed to infection.^35^ In particular, *Bifidobacterium pseudolongum* and *Bifidobacterium animalis* levels were significantly elevated in surviving mice when compared to mice which died. It was suggested that *Bifidobacterium* mediates the anti-influenza effect via several specific metabolic molecules including valine and coenzyme A.^35^

**Table 1:** Changes of the gut microbiota composition following influenza infection.

Taxonomy	Variation after infection	Subtype virus	Samples	References
Proteobacteria phylum	↗	H1N1	F	^45,47^
* Betaproteobacteria* class (Sutterella genus)	↘	H3N2, H1N1	F, C	^47^
* Gammaproteobacteria* class	↗	H5N1	SM	^49^
* Escherichia* genus	↗	H3N2, H1N1	F, C, SM	^45,47,48^
* Enterobacteriaceae* order	↗	H1N1	F, SM	^45,48^
Bacteroidetes phylum	↘	H5N1	SM	^49^
* S24-7* family	↘	H3N2, H1N1, H5N1	F, C, SM	^47,49^
	↗	H1N1	F	^46^
* Porphyromonadaceae* family	↗	H3N2, H1N1	F, C	^46,47^
* Bacteroidia* class	↘	H5N1	SM	^49^
Firmicutes phylum	↗	H5N1	SM	^49^
* Bacilli* class	↗	H5N1	SM	^49^
* Lactobacillus* genus	↗	H5N1	SM	^49^
	↘	H3N2, H1N1	F, C, SM	^47,48^
* Clostridiales* family (unaffiliated)	↗	H3N2, H1N1	F, C	^47^
* Ruminococcaceae* family	↗	H3N2	F, C	^47^
* Ruminococcus* genus	↗	H3N2, H1N1	F	^47^
* Gemmiger* genus	↗	H3N2	F	^47^
* Mogibacteriacecea* family	↗	H3N2	F, C	^47^
* Coprococcus* genus	↗	H3N2	F	^47^
* Roseburia* genus	↗	H3N2	F	^47^
* Defluvittalea* genus	↗	H3N2	F, C	^47^
* Dorea* genus	↗	H3N2	F	^47^
* Segmented Filamentous Bacteria* genus	↘	H1N1	F, SM	^45,48^
Cyanobacteria phylum	↗	H3N2, H1N1	F	^47^
Verrucomicrobia phylum	↗	H3N2, H1N1	F, C	^44,47^

*F* fecal sample, *C* cecal sample, *SM* small intestine.

Surprisingly, few studies have analysed the nature of gut microbiota alteration during viral respiratory infections in the human setting. Quin and colleagues analysed samples from 40 patients infected with H9N2 avian virus.^51^ A decrease in diversity and the overgrowth of *Escherichia coli* and *Enterococcus faecium* were observed. *Eubacterium*, *Ruminococcus*, *Bifidobacterium*, and *Roseburia* were all less abundant in infected patients. In a cohort of 24 influenza A (H1N1) patients, Gu et al. reported a decrease in the relative abundance of Actinobacteria, Erysipelotrichea, Clostridia and beneficial butyrate producers (*Lachnospiraceae* and *Ruminococcaceae* families).^52^ On the other hand, opportunistic pathogens such as *Shigella* and *Escherichia* species developed. Studies of the impact of influenza infection on the gut microbiota in large human cohorts are now warranted.

Gut microbiota’s metabolites are important in health. Whether or not change in the gut microbiota composition during IAV infection alters the gut’s metabolic output has been recently addressed. In the mouse system, infection with IAV led to changes in the gut (cecal) metabolome, with a significant drop in the production of SCFAs.^47^ The concentrations of acetate, propionate, and butyrate were all lower than in mock-infected controls. Infection with IAV also resulted in a shift in glycolipid metabolism. Interestingly, IAV infection altered the synthesis of alpha-galactosylceramides^53^ which are known ligands for invariant natural killer T cells and play an important role in the immune system in rodents and humans.^54^ It is not clear whether these metabolic changes influence gut homeostasis and function. However, one can speculate that reduced alpha-galactosylceramide availability may interfere with the immune regulatory functions of intestinal invariant natural killer T cells.^55^ Along with ligands for (invariant) natural killer T cells, it is also possible that the synthesis of ligands for mucosal-associated invariant T (MAIT) cells could also be altered during a viral respiratory infection. Indeed, bacterial commensals, including species belonging to Bacteroidetes and Proteobacteria phyla, as well as pathogenic bacteria, can produce vitamin B2 and vitamin B9 metabolites, known to act as agonists or antagonists for MAIT cells. Regarding the key role of MAIT cells in mucosal immunity,^56–58^ altered synthesis of MAIT cell ligands during viral respiratory infections might have important consequences on disease outcomes, a hypothesis that needs to be investigated.

### Respiratory syncytial virus and gut microbiota

RSV is the most common cause of bronchiolitis and pneumonia in children under the age of 2 years. It accounts for over 80% of pediatric lower respiratory tract infections.^59^ This pathogen is also a significant cause of respiratory illness in older adults. As observed with IAV infection, RSV induces seasonal outbreaks and causes up to 118,000 deaths each year worldwide.^60^ In a mouse model, RSV resulted in significant alterations in gut microbiota diversity (but not abundance and alpha diversity) at day 7 post infection, with an increase in Bacteroidetes and a decrease in Firmicutes.^46^ This increase in the Bacteroidetes phylum was mainly due to the rise in the *Bacteroidaceae* and *S24-7* family, whereas the decreased abundance of Firmicutes was related to attenuation of both *Lachnospiraceae* and *Lactobacillaceae* families. The impact of RSV infection on the gut microbiota’s metabolic activity has rarely been addressed. In one study, RSV infection predominantly increased the metabolism of lipids, including sphingolipids, polyunsaturated fatty acids, and the SCFA valerate.^61^

### Coronaviruses and gut microbiota

Coronaviruses continuously circulate in human populations and generally cause mild respiratory diseases, including the common cold. In contrast, SARS-CoV and the Middle East respiratory syndrome coronavirus—both of which are zoonotic in origin—can cause severe respiratory diseases and have a high mortality rate.^62^ Specific antivirals or approved vaccines against SARS-CoV and Middle East respiratory syndrome coronavirus are not yet available. In late 2019, a new infectious respiratory disease emerged in Wuhan, China.^63,64^ This disease (now termed COVID-19, caused by SARS-CoV-2) rapidly spread through China and many other countries worldwide.^64^

Surprisingly, few studies have addressed the impact of coronavirus infection on the gut microbiota and most of them focused on chicken and pig models (induction of microbial imbalance).^65–69^ Recent studies have reported the impact of a SARS-CoV-2 infection on the gut microbiota in humans.^52,70–73^ Briefly, SARS-CoV-2 infection lowered the abundance of butyrate producers such as several genera from the *Ruminococcaceae* and *Lachnospiraceae* (*Roseburia*) families.^71^ A significantly higher relative abundance of opportunistic bacterial pathogens including *Streptococcus* (class Bacilli), *Rothia*, and *Actinomyces* was also observed. Another recent study revealed, by RNA shotgun metagenomics sequencing, the presence of opportunistic bacterial pathogens including *Collinsella aerofaciens* and *Morganella morganii* as well as *Streptococcus infantis* (an abundant colonizer in the upper respiratory tract) in fecal samples of patients with COVID-19 who had high SARS-CoV-2 infectivity.^73^ In contrast, SCFAs and tryptophan producers were enriched in the fecal samples with signature of low-to-none SARS-CoV-2 infectivity. Of interest, the feces with high SARS-CoV-2 infectivity had a higher microbiome functional capacity for nucleotide de novo biosynthesis, amino acid biosynthesis and glycolysis.^73^ Of note, overgrowth of opportunistic fungal pathogens (*Aspergillus* and *Candida* spps) was also described in COVID-19 patients.^72^ It is interesting to notice that in the Gu’s study, similarities and also differences between influenza (H1N1) and SARS-CoV-2 patients were described.^52^ The discovery of core gut microbial features—and related metabolites—may serve as potential diagnostic markers.

### Perspectives

Animal models and clinical data clearly show that acute viral respiratory infections lead to a transient disruption of the balanced gut microbial ecosystem, with less salutary bacteria and more pathogenic bacteria. However, a more comprehensive description must be obtained. Further research should include metagenomics analyses such as shotgun metagenomic sequencing. The later approach offers the possibility to analyse potential changes in the composition of fungi, viruses and parasites and to evaluate major changes in the microbial metabolic pathways.^74^ In parallel, large human cohorts and more relevant animal models (e.g., in nonhuman primates) are needed. These approaches will be instrumental in determining the potential clinical relevance of the findings. Along the same line, a better understanding of the overall impact of acute viral respiratory infection on the gut microbiota’ metabolic output is urgently needed. Research in this area is in its infancy in the context of viral respiratory infections. Metabolomics studies might provide a more detailed understanding of consequences of gut microbiota changes on disease outcomes and might also lead to the discovery of biomarkers of disease severity. Genome-scale metabolic modeling will also be instrumental to estimate the metabolic capabilities of gut microbiota. Correlation analyses between changes in gut microbiota composition, microbial metabolites, proinflammatory cytokine levels and disease severity will be very informative. This approach may serve as a preventive/treatment target for intervention especially among patients who are highly susceptible to viral respiratory infections.

## Mechanisms leading to changes in gut microbiota composition during viral respiratory infections

**Fig. 2:**
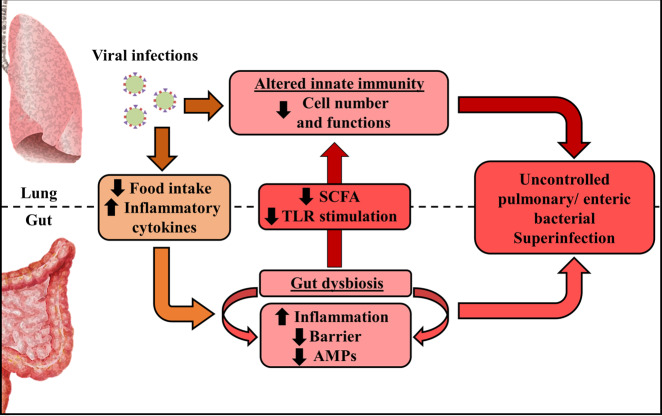
The gut–lung axis during viral respiratory infections. Viral respiratory infections, such as influenza, alter the functions of pulmonary immune and stromal (epithelial) cells, thus leading to secondary bacterial infections. Meanwhile, systemic inflammatory cytokines, inappetence (less fiber), change in oxygen levels, and altered epithelial metabolism disrupt the composition and function of intestinal microbiota. These alterations contribute to intestinal inflammation, epithelial barrier disruption, and decreased production of antimicrobial peptides (AMPs). Secondary enteric infections then develop. Epithelial leakage may enhance bacterial translocation and trigger systemic inflammation and organe dysfunction. Dysbiosis due to viral respiratory infection also results in diminished production of microbial-associated molecular patterns including Toll-like receptor (TLR) and nucleotide oligomerization domain (NOD)-like agonists and microbial metabolites such as SCFAs, thus reducing antibacterial pulmonary immunity. Hence, by altering the gut homeostasis, respiratory viral infections lead to bacterial superinfection. The interconnectedness of the lung and the gut might be particularly relevant during SARS-CoV-2 infection.

There are several causes of gut microbiota changes during acute viral respiratory infections; these may include the release of inflammatory cytokines and reduced food intake (Fig. 2). In the mouse, infection with IAV or RSV induces substantial weight loss, ranging from 10 to 20% of the initial weight and peaking at days 5–7 post infection.^47,61,75,76^ This weight loss is mainly due to a loss of appetite (inappetence).^77,78^ Reductions in food and calorie intakes are known to perturb the gut microbiota.^79^ The results of pair-feeding experiments have clearly indicated that a rapid fall in food intake mimics the changes in the gut microbiota observed during infection (i.e., enhanced abundance of Verrucomicrobia phylum, alphaproteobacteria class, and *Parabacteroides* Genus and reduced abundance of *Lachnospiraceae*, *Ruminococcus*, and *Lactobacillus* genus) although differences (*Clostridiales* Family) were also observed.^47,61^ Consistently with the decrease in fiber intake, pair-fed mice had lower SCFA concentrations.^47^ Among them, butyrate fuels colonic epithelial cells and favors epithelial oxygen consumption, thus allowing oxygen deprivation in the intestinal lumen. The drop of SCFAs due to reduced food (fiber) intake is likely to alter epithelial cell metabolism. One of the main explanation for inappetence during viral infection is the overproduction of inflammatory cytokines, including tumor necrosis factor alpha.^80^ The neutralization of this cytokine during an RSV infection reduced the weight loss and partly attenuated the perturbation of the gut microbiota.^61^ Along the same line, depleting cytokine-producing CD8^+^ T cells during an RSV infection reduced inappetence and reversed changes in the gut microbiota.^61^ Type I and II IFNs are essential components of the host antiviral response - notably during influenza.^81^ These cytokines potently disturbed the gut microbiota.^45,48^ Relative to wild type mice, mice deficient in type I IFN receptor presented reduced blooming of Proteobacteria (*Escherichia* genus) and, conversely, an elevated proportion of segmented filamentous bacteria.^45^ Along the same line, depletion of IFN-γ during an IAV infection restored changes in segmented filamentous bacteria (reduced in IFN-γ-competent mice), the *Lactobacillus* genus (reduced), and *Enterobacteriaceae* (augmented).^48^ Hence, inflammatory cytokines and loss of appetite can drive gut dysbiosis during viral respiratory infections, at least in the mouse system. Other mechanisms might also be implicated. Intestinal inflammation, due for instance to infiltrated CD4^+^ T cells or systemic IFN release,^45,48^ is known to alter the metabolism of epithelial cells—resulting in the accumulation of a novel set of nutrients, for which the microbes that inhabit the intestinal lumen compete. Along with increased oxygen availability (see below), this phenomenon appears to explain the shift from obligate anaerobes to facultative anaerobes such as Proteobacteria *Enterobacteriaceae.*^82–86^ Hypoxia is a major clinical symptom during the acute phase of respiratory viral infection, including in COVID-19 patients.^87^ It also associates with chronic intestinal injury. Regarding the role of oxygen in intestinal homeostasis, including microbiota composition and function,^88^ disruption of the oxygen gradient for instance due to SCFA availability, might play a part in gut dysbiosis and gastrointestinal disorders during respiratory viral infections. Defective intestinal (epithelial) immune functions during respiratory viral infections (reduced production of antimicrobial peptides) may also participate in dysbiosis.

In contrast to the situation with IAV and RSV, viral RNA is detected in the gut during coronavirus infection, including SARS-CoV-2 infection.^89–92^ Around half of COVID-19 patients have SARS-CoV-2 RNAs in the stools—even when it is no longer found in the respiratory tract.^90–92^ Importantly, infectious viruses were detected in fecal samples of COVID-19 patients suggesting that the digestive tract might be a site of viral replication and activity.^93,94^ In line, several recent studies using human small intestinal organoids have shown that SARS-CoV-2 replicates in enterocytes.^93,95,96^ Hence, local viral replication is likely to disturb the local ecosystem leading to changes in the gut microbiota composition and functions. Moreover, binding of the viral spike protein to the cell surface receptor angiotensin-converting enzyme II (ACE2) results in downregulated expression of the latter.^97^ With regard to the critical role of ACE2 in the maintenance of the gut’s microbial ecology (via malfunction of amino acid transport, tryptophan deficiency and decreased production of antimicrobial peptides),^98^ it is likely that the lack of available ACE2 during a SARS-CoV-2 infection has a critical role in dysbiosis.

## Impact of respiratory virus-induced changes of the gut microbiota on disease outcomes

Fecal transfer experiments indicated that a gut microbiota collected from mice that have survived influenza (H7N9) infection can transfer protection in recipient (naive) mice challenged with IAV.^35^ Whether this protective effect extends to other IAV subtypes and to other respiratory viruses is still unknown and worth of future studies. Hence, alteration of the gut microbiota composition and functional activities due to respiratory viral infections might lead to beneficial effects. However, as developed below, dysbiosis due to an acute respiratory viral infection also leads to detrimental effects and participates in the outcomes of infection.

### Consequences on gut homeostasis

The gut microbiota is critical in the maintenance of epithelial integrity and in the development of regulatory T cells.^1–8^ This critical role depends mostly on microbiota’s metabolites. During homeostasis, the gut exerts a relatively anti-inflammatory immune state.^99^ Perturbations of the gut microbiota contribute to several gut diseases, such as inflammatory bowel disease, irritable bowel syndrome, coeliac disease, and colorectal cancer.^100,101^ Based on these observations, researchers have investigated the possible negative impact of the gut dysbiosis observed during viral respiratory infections on gut homeostasis and functions.

As stated above, viral respiratory infections, including IAV, RSV, and coronavirus infections, can induce gastroenteritis-like symptoms, such as abdominal pain, nausea, vomiting, and diarrhea.^43,102,103^ Mice infected with IAV presented intestinal injuries, including a reduction in the length of the colon (a marker of inflammation), elimination of the mucosal layer in the small intestine, and mild diarrhea.^48,49^ In parallel, enhanced mRNA expression of inflammatory cytokines and IFN-stimulated genes was observed.^45,48^ One important feature of the gut is its barrier function; under healthy conditions, this prevents the excessive diffusion of microbial components. Many diseases are associated with disruption of the barrier function and thus lead to bacterial translocation, systemic inflammation, and shock. The impact of IAV on the gut’s barrier property has not been characterized in details^104^ and merits investigation. In the case of RSV, no histological evidence of significant inflammation of the colon was observed in a mouse model, other than an elevated feces level of lipocalin-2, a marker of gut inflammation.^46^

One can question whether in the context of viral respiratory infection, the altered microbiota initiates gut inflammation, or whether inflammation induces dysbiosis. This is a “chicken and egg” situation. As stated above, inflammation might influence the composition of the gut microbiota. On the other hand, the dysbiotic microbiota might directly initiate and sustain gut disorders. Indeed, antibiotic treatment during an IAV infection (to limit the increase in *Enterobacteriaceae*) reduced intestinal inflammation.^48^ Moreover, fecal transfer experiments indicated that the IAV-experienced microbiota triggered the local accumulation of T helper 17 cells, which subsequently cause gut inflammation.^48^ Along with *Enterobacteriaceae*, it is also likely that mucus-degrading bacteria (such as *Ruminococcus* and *Akkermansia*) are important factors in the maintenance and/or amplification of gut inflammation. Indeed, these bacteria are known to erode the colonic mucosa and favor interactions between luminal bacteria and the intestinal epithelium; in turn, this leads to inflammation and the impairment of barrier function.^105,106^

Taken as a whole, viral respiratory infections influence gut homeostasis—possibly via a change in the equilibrium of commensals. The nature of the intestinal disorders and the infection’s consequences on gut physiology (e.g., the digestion and absorption of ingested foods and liquids), metabolism and immune homeostasis remain to be analysed in details. Altered gut function following viral respiratory infections might have both immediate and long-term consequences for health. For example, the gut barrier is vital for the maintenance of homeostasis, and any perturbation can lead to the systemic dissemination of bacterial components and thus harmful health outcomes.

### Consequences on secondary enteric infections

The gut microbiota is vital in the local control of pathogenic infections through direct microbial antagonism and stimulation of the host’s effector responses (e.g., antimicrobial peptides).^107,108^ Dysbiosis can increase the risk of developing an enteric bacterial infection.^109,110^ Experiments in animal models showed that IAV enhances susceptibility to secondary enteric infection by *Salmonella enterica* serovar Thyphimurium.^45,49^ This enhancement was due, at least in part, to type I IFNs and the associated reduction in antimicrobial peptide and inflammatory cytokine release.^45^ Reduction in the gut microbiota’s diversity is also likely to be involved in secondary enteric infection;^49^ for instance (in the mouse system), the proportion of segmented filamentous bacteria (known to be important in T helper 17-mediated immune responses) fell during IAV infection.^50^ In parallel, a fall in the proportion of SCFA producers^47^ might be important in secondary enteric infections. These fatty acids are known to control the equilibrium of the gut microbiota and to prevent the development of enteric pathogens like *Enterococcus faecalis* and *Salmonella enterica* serovar Thyphimurium.^111,112^ It remains to be seen whether oral supplementation of SCFAs during IAV infection can lower secondary enteric infection.

### Consequences on pulmonary bacterial superinfections

One of the major complications of respiratory viral infections is increased susceptibility to secondary bacterial infection—mainly those induced by *Streptococcus pneumoniae*, *Staphylococcus aureus*, and *Haemophilus influenzae*. This complication primarily affects children and older adults^113,114^ and contributes to the excess morbidity and mortality observed during epidemics and pandemics.^42^ Experiments in animal models have demonstrated that the enhanced susceptibility is due in part to an impairment of the antibacterial innate immune response (Fig. 2).^42,115^

A healthy gut microbiota has a beneficial, physiological role in pulmonary immunity,^12,13^ including a positive role in controlling the development of opportunistic bacteria such as by *S. pneumoniae* or *S. aureus.*^19,21,23,47,116^ We have recently investigated the possibility that perturbation of the gut microbiota during influenza infection could increase the incidence of bacterial superinfection in lungs. Indeed, fecal transfer experiments demonstrated that the IAV-conditioned microbiota compromised the lung’s defenses against pneumococcal infection (Fig. 2).^47^ In mechanistic terms, a fall in the production of acetate (the predominant SCFA produced by the microbiota) affected the bactericidal activity of alveolar macrophages. In the context of dual influenza and pneumococcal infections, acetate supplementation lowered bacterial loads, reduced lung disease, and enhanced survival rate. Hence, changes in gut microbiota (and ablated SCFA production) during influenza is associated with bacterial superinfection. Further research in this field might help to define predictive markers (e.g., systemic SCFAs) and/or to develop therapeutic approaches against bacterial superinfections, for instance by harnessing the power of the gut microbiota via prebiotics and/or probiotics. Of note, along with the gut microbiota, it is also likely that changes in microbial composition and function in the respiratory tract, that associate with acute viral respiratory infections (for a review,^117^), are important in bacterial superinfection. This local dysbiosis may alter the dynamics of inter-microbial interactions, thereby enhancing the proliferation of potentially pathogenic bacterial species. Changes of local microbial metabolic output might also lower pulmonary defense against secondary bacterial infection. Collectively, gut dysbiosis during viral respiratory infections (at least influenza) impacts on bacterial superinfection in lungs. Whether microbial changes affect other disease outcomes during viral respiratory infections such as ARDS, sepsis and multiple organ dysfunction is still an open question.

### The case of SARS-CoVs

The conseqences of the gut dysbiosis on COVID-19 progression and severity has yet to be fully characterized. COVID-19 patients with gastrointestinal symptoms have overall more severe/critical diseases indicating the importance of the lung–gut axis in this setting.^118,119^ The available evidence suggests that SARS-CoV-2 infection alters the gut barrier, leading to the systemic spread of bacteria, endotoxins, and microbial metabolites.^63,120,121^ This might affect the host’s response to COVID-19 infection and might contribute to multisystem dysfunction, septic shock, and the systemic inflammatory storm that occurs in the second phase of the SARS-CoV-2 infection and which is in part responsible for the disease’s mortality. Gut disorders during SARS-CoV-2 infection might also participate in concomitant or secondary bacterial infections, which develop in severely ill COVID-19 patients.^122–124^ Gastrointestinal disorders in patients with COVID-19 are associated with a more aggressive clinical course, including ARDS, liver injury, a higher body temperature, and shock.^118^ The nature of the intestinal disorders, particularly the microbiota’s function and the gut’s barrier property, must be urgently investigated, with a view to developing targeted therapies. Risk factors for COVID-19 (e.g., aging, and metabolic diseases such as obesity and diabetes) may be of particular importance.^125^ Indeed, obesity and diabetes are known to be associated with disturbances of the intestinal microbiota and an impairment of the gut’s barrier function.^6,36,37^ When combined with the virus’s effect on gut homeostasis, these chronic impairments might amplify the severity of COVID-19.

## Conclusion

The gut microbiota is vital in the lung’s defenses against respiratory infections, as exemplified by the cases of IAV and RSV infections. It remains to be seen whether this is also the case for other dangerous respiratory viruses, such as SARS-CoV-2. The use of relevant animal models should rapidly generate important information for the design of interventional strategies that can reinforce the lungs against viral pathogens. These strategies might include the use of SCFA-producing probiotics and prebiotics, such as fiber-rich diets. These procedures might reinforce the gut microbiota’s ability to preventively arm the immune system and also protect the microbiota against the perturbations associated with viral infections. Alteration of the gut microbiota not only participates in intestinal disorders but also favors systemic damage and bacterial superinfections. Research in this area may lead to preventive and therapeutic approaches to better fight against viral respiratory infections.
